# ^1^H, ^15^N and ^13^C assignment of the amyloidogenic protein medin using fast-pulsing NMR techniques

**DOI:** 10.1007/s12104-015-9641-z

**Published:** 2015-09-16

**Authors:** H. A. Davies, M. M. Phelan, J. Madine

**Affiliations:** Institute of Integrative Biology, University of Liverpool, Crown Street, Liverpool, L69 7ZB UK

**Keywords:** Medin, Amyloid, Fast-pulsing NMR, Resonance assignment, Cardiovascular, Fibril

## Abstract

Thirty-one proteins are known to form extracellular fibrillar amyloid in humans. Molecular information about many of these proteins in their monomeric, intermediate or fibrillar form and how they aggregate and interact to form the insoluble fibrils is sparse. This is because amyloid proteins are notoriously difficult to study in their soluble forms, due to their inherent propensity to aggregate. Using recent developments in fast NMR techniques, band-selective excitation short transient and band-selective optimized flip-angle short-transient heteronuclear multiple quantum coherence we have been able to assign a 5 kDa full-length amyloidogenic protein called medin. Medin is the key protein component of the most common form of localised amyloid with a proposed role in aortic aneurysm and dissection. This assignment will now enable the study of the early interactions that could influence initiation and progression of medin aggregation. The chemical shifts have been deposited in the BioMagRes-Bank accession Nos. 25399 and 26576.

## Biological context

Medin is the principal protein component of the most common form of localised amyloid and is thought to have a role in aortic aneurysm and dissection. Despite the prevalence of these amyloid plaques, medin is a relatively poorly understood protein. Medin is proposed to derive from the cleavage of the milk-fat globule protein MFG-E8. The medin sequence is located within the C2 domain of MFG-E8 but the mechanism for excision is not understood (Häggqvist et al. 1999). Within the PDB there are three deposited structures of the C2 domain of MFG-E8; two bovine structures determined by X-ray crystallography (2PQS and 3BN6) and a murine structure determined by solution state NMR (2LDL). There is only 66 % sequence identity between murine and human and 69 % between bovine and human C2 domains. To our knowledge there are no assignments or structures of the human form of MFG-E8 or any isolated domain structures. Previous biophysical measurements of medin at 20 μM concentration indicated that fibrillisation occurred after approximately 30 h (Davies et al. 2014, 2015). Traditional NMR assignment methods typically require high protein concentrations. Due to the nucleation dependent growth of amyloid proteins, increased protein concentration can reduce the lag time for fibrillisation (Harper and Lansbury 1997), making time-consuming assignment experiments of these proteins in their soluble form prohibitive. As an alternative we sought to assign medin under denaturing conditions (8 M Urea) and subsequently transfer the assignment to spectra collected on the initial pre-fibrillar native form. This enabled an increase in protein concentration and reduced peak-width, essential for collecting the less sensitive triple resonance NMR experiments. Additionally the use of fast pulsing (BEST) multidimensional NMR experiments enabled triple resonance experiments to be collected in a fraction of the time.

## Methods and experiments

**Fig. 1:**
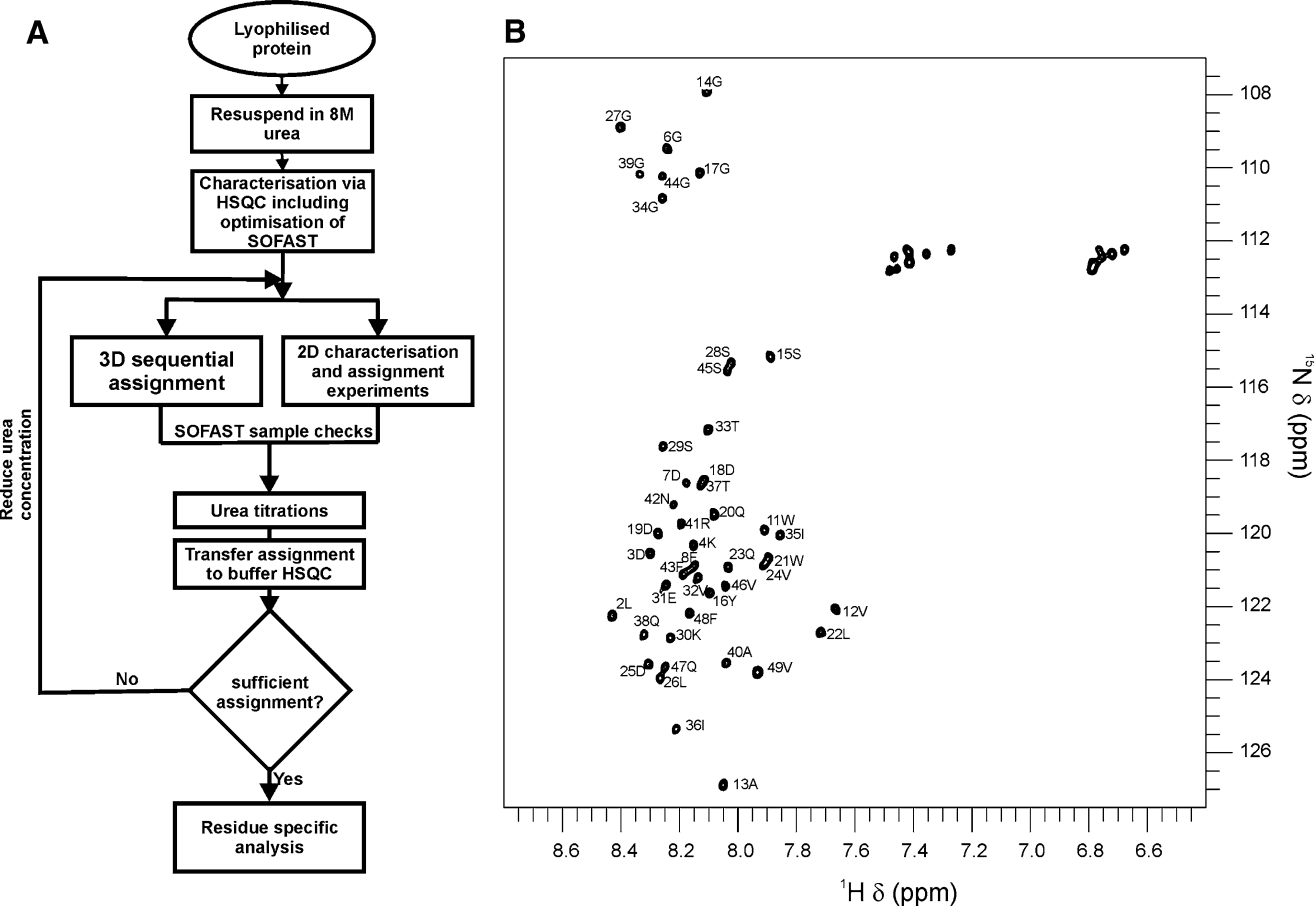
NMR assignment of medin. An iterative process was undertaken to achieve assignment of medin in a physiological buffer, utilising denaturing conditions and fast-pulsing NMR techniques (**a**). Assigned ^1^H–^15^N HSQC for medin in a physiological buffer (20 mM sodium phosphate, 20 mM NaCl, pH 6.5) (**b**). Spectrum collected with 40 μM ^15^N uniformly labelled medin sample

^13^C, ^15^N isotope labelled medin were expressed as previously described (Davies et al. 2014) with the substitution of Lemo 21 (DE3) cells. Cell pellets were resuspended in 6 M guanidine hydrochloride (GdmCl), 20 mM sodium phosphate, 0.5 M NaCl, pH 8.0 and frozen at −20 °C. Cells were thawed, homogenised, and cell debris removed by centrifugation (19,000*g*, 15 min, 4 °C). The supernatant was loaded onto a 5 ml Ni^2+^–NTA column and washed with 4 column volumes (CV) of 6 M GdmCl, pH 8, followed by 4 CV of 6 M GdmCl, pH 6, and eluted with 3 CV of 6 M GdmCl, pH 2, and stored at −20 °C. Fusion protein was buffer exchanged into 20 mM Tris–Cl, 0.15 mM NaCl, pH 7.4, and the His6-SUMO tag was removed by incubation with SUMO protease I at 4 °C for 3 h. The cleavage mixture was then passed through a 5 ml Ni^2+^–NTA column and the flow through containing medin collected. Medin was buffer exchanged into d dH_2_O and lyophilised. The lyophilised protein was then resuspended in 20 mM sodium phosphate, 20 mM NaCl, pH 6.5 with or without the addition of 8 M urea for NMR assignment. For triple resonance assignment medin was reconstituted to a concentration of 200 μM in 8 M urea with 10 % (v/v) ^2^H_2_O. Spectra were acquired at 25 °C on Bruker AVANCE III 600 and 800 MHz spectrometers equipped with 5 mm triple resonance (TCI) cryoprobes. A schematic of the experimental workflow is detailed in Fig. 1a. In brief, 2D SOFAST-HMQC and 3D BEST-experiments (HNCO, HNCACO, HNCOCACB, HNCACB) were recorded for ^1^H_N_, ^13^Cα, ^13^Cβ ^13^C′ and ^15^N_H_ assignments. Spectra were assigned in 8 M urea and the N_H_ and H_N_ backbone assignment then transferred using a series of 2D HSQC experiments collected at 6, 4, 2, 1 and 0 M urea. Spectra were processed using Topspin 3.1 (Bruker) and assignment carried out using CCPN Analysis (Vranken et al. 2005). SOFAST-HMQC experiments were interleaved throughout to assess sample stability.

## Assignment and data deposition

^1^H–^15^N HSQC spectrum exhibited peaks consistent with the protein being predominantly unfolded and gave discrete peaks of limited dispersion. In 8 M urea, medin remained in solution for at least 60 h at 200 μM. 3D sequential assignment experiments were performed using BEST experiments to increase the number of scans attainable during the stable phase of the protein (Schanda et al. 2006; Lescop et al. 2007). As a result, a complete ^15^N, ^1^H_N_, assignment of the stable denatured state was achieved (excluding N and C-terminal residues), along with over 89 % for backbone atoms ^13^CO, ^13^Cα, ^13^Cβ. The corresponding chemical shifts have been deposited in the BioMagRes-Bank; Accession No. 25399. A urea titration series (8–0 M) was used to transfer assignments to a ^1^H–^15^N HSQC spectrum with 0 M urea, the corresponding chemical shifts have been deposited in the BioMagRes-Bank; Accession No. 26576. To remove ambiguity in some peak assignments, BEST experiments were also carried out in 4 M urea. The interleaved SOFAST-HMQC data were acquired in 8 min (Schanda and Brutscher 2005; Schanda et al. 2005). Triple resonance spectra were acquired for soluble medin in 8 M urea in 26 h with resolution in the indirect dimension high enough to resolve all unfolded signals. Urea titration enabled transfer of NH assignment to the native form resulting in 94 % of backbone NH shifts assigned in 0 M urea (Fig. 1b). Chemical shift changes from 8 M to 0 M urea were systematic and rarely deviated in the ^15^N dimension indicative of minimal conformational changes and also suggesting that medin has a predominantly random coil structure in 0 M urea.

This method of assignment transfer utilizing a combination of fast pulsing techniques and denaturants sufficiently extended the duration of protein in the soluble phase (although it should be noted the presence of denaturant did not halt the process of aggregation completely). We envisage that this method of assignment can be used for other rapidly aggregating proteins that would be otherwise intractable to NMR assignment in the soluble phase.
